# Ceramide Synthases Are Attractive Drug Targets for Treating Metabolic Diseases

**DOI:** 10.3389/fendo.2020.00483

**Published:** 2020-07-29

**Authors:** Suryaprakash Raichur

**Affiliations:** Evotec International GmbH, Göttingen, Germany

**Keywords:** ceramides, sphingolipids, C16 ceramide, insulin resistance, obesity, NAFLD, NASH and mitochondrial dysfunction

## Abstract

Ceramide synthases (CerS) are central enzymes required for the *de-novo* synthesis of ceramides and other sphingolipids. They catalyze the addition of different acyl-chains to a sphingoid base, and thus account for much of the rich diversity in the sphingolipid family. Recent studies have demonstrated that the acyl-chain is an important determinant of ceramide function, such that a small subset of ceramides (e.g., those containing the C16 or C18 acyl-chain) alter metabolism by inhibiting insulin signaling or inducing mitochondrial fragmentation. Herein I discuss the therapeutic potential of targeting certain ceramide synthase isoforms for the treatment of obesity, insulin resistance, steatohepatitis, and other metabolic disorders.

## Introduction

The increasing burden of metabolic diseases such as diabetes and heart disease is alarming, not only in developed countries, but throughout the world. Some prevalent metabolic disorders, such as non-alcoholic steatohepatitis (NASH), have no approved pharmacotherapies (1, 2). Unhealthy lifestyles fuel these pathologies, as combinations of sedentary lifestyles and poor dietary habits promote the delivery of excess saturated fatty acids and carbohydrates into non-adipose tissues such as liver and skeletal muscle, impairing their function (3, 4). Numerous studies reveal that the conversion of these excess fuels into sphingolipids such as ceramides is a critical event that leads to the cellular defects that accompany obesity (5, 6).

Clinical studies confirm that excess saturated fatty acid intake elevates levels of serum and tissue ceramides (7). Contrastingly, polyunsaturated fat intake reduces serum ceramides (7). In rodents, inhibiting ceramide biosynthesis using genetic and pharmacological approaches ameliorates atherosclerosis, hepatic steatosis, insulin resistance and obesity (8).

**Figure 1:**
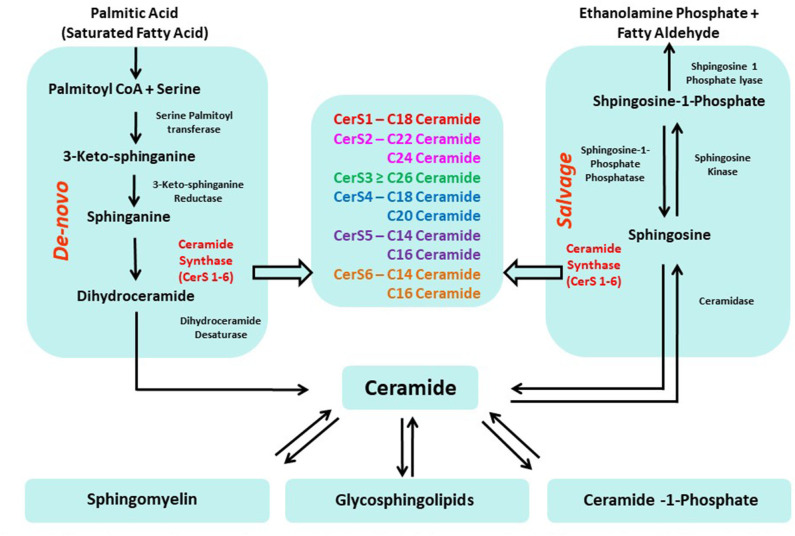
Two primary pathways produce ceramides in cells: (a) *de novo* synthesis (b) re-acylation of sphingosine (salvage pathway).

Two primary pathways produce ceramides in cells (Figure 1): (a) a *de novo* synthesis enzyme cascade that starts with the condensation of a saturated acyl-CoA (typically palmitoyl-CoA) and amino acid (typically serine) to produce the sphingoid backbone; and (b) a salvage pathway that involves the re-acylation of sphingosine. In both cases, ceramides (or dihydroceramides, in the case of the *de novo* synthesis pathway) are produced by ceramide synthases (CerS) through N-acylation of the sphingoid base. Mammalian CerS exists in six isoforms (CerS1-6) with differing preferences for specific fatty acid chain lengths. CerS1 attaches C18 fatty acyl CoA to the sphingoid base; CerS2 attaches very long fatty acyl CoAs such as C22–C24; CerS3 attaches C26–C34 acyl CoA; CerS4 attaches C18–C20 fatty acyl CoA; and CerS5 and 6 have specificity for C14–C16 fatty acyl CoA. Thus, the CerS enzymes determine the acyl-chain composition of ceramides. CerS expression, structure, localization, knock-out phenotypes, and association with other diseases have been reviewed comprehensively (9).

Ceramides can be further modified by the addition of different head groups, and the number of estimated bioactive sphingolipids range from 4,000 to 60,000 (10). The ceramides themselves have emerged as important signaling molecules that contribute to cellular stress responses (11). A primary mechanism through which ceramide promotes insulin resistance is by decreasing the activity of Akt/PKB, which is an essential facilitator of cellular glucose uptake. Ceramide blocks the activity of Akt/PKB by independent mechanisms: by enhancing Akt dephosphorylation via protein phosphatase 2A (PP2A) and by blocking the translocation of Akt via PKCz (12). Other ceramide actions have been identified that contribute to triglyceride production, mitochondrial dysfunction, and ultimately apoptosis (13).

Several genetic and pharmacological studies targeting *de novo* ceramide biosynthesis focused on the first enzyme in the pathway: serine palmitoyltransferase (SPT). Inhibiting this enzyme in rodents reduces global ceramide levels and ameliorates many forms of metabolic diseases (14). However, SPT is not a viable therapeutic target, owing to safety issues that result from the extreme diminution of all sphingolipids (15, 16). Therefore, therapeutic approaches focusing on a more limited subset of detrimental ceramides subspecies may represent a new strategy for therapeutic intervention. Fortunately, two recent reports demonstrated that the sphingolipid acylation patterns influences disease progression (17, 18), thus suggesting that the CerS enzymes might be viable targets.

## Clinical Correlations Between Specific Ceramides and the Metabolic Syndrome in Humans

Studies in clinical cohorts reveal striking relationships between serum or tissue ceramides and various measures of cardiometabolic disease. However, considerable variability exists regarding the precise sphingolipids that are most commonly elevated in a diseased individual. In the following paragraphs, I will focus on the larger studies that reveal specific roles for certain ceramides in diabetes and NASH.

### Insulin Resistance and Diabetes

Insulin resistance, a condition typically defined as an inability of insulin to appropriately clear glucose from the bloodstream, is a risk factor for diabetes, heart disease, and NASH. Clinical observations reveal that circulating ceramides packaged in LDL (low-density lipoprotein) negatively associate with insulin sensitivity (19). The authors also demonstrated that infusion of LDL-containing ceramides into healthy mice attenuated whole body glucose clearance and increased levels of inflammatory markers (19), thus confirming that circulating lipids can be taken into tissues to alter metabolism. These authors also demonstrated that LDLs containing C16:0 and C24:0 ceramides reduced glucose uptake in cultured myotubes by inhibiting insulin signaling and decreasing translocation of the GLUT4 glucose transporter (19). In the Dallas Heart Study, consisting of 1,557 participants without type 2 diabetes assessed for metabolic biomarkers, fat depots and plasma ceramides over a period of 7 years, the saturated C16 and C18 ceramides correlated with insulin resistance (i.e., homeostatic model assessment of insulin resistance, HOMA-IR), total body fat and visceral adipose tissue (20). In contrast, healthier metabolic profiles were associated with longer-chain polyunsaturated fatty acid ceramides C24:2, C30:10, and C32:11 (20). Similarly, an evaluation of plasma sphingolipids in a large cohort of Chinese individuals in Singapore revealed that C16, C18, and C20 ceramides containing a d16:1 backbone correlated positively with body mass index (BMI) and HOMA-IR (21). By comparison, hexosylceramides and ceramides with a d18:2 backbone negatively correlated with HOMA-IR and BMI (21). Off note, this study comprehensively assessed both the d16:1 and d18:2 backbone of sphingolipid species, whereas most of the studies generally focus on most abundant d18:2 backbone (21). Another interesting clinical study investigated ceramide subspecies and their ratios to determine the best predictors of diabetes (22). The authors identified C18/16 ratios as an independent marker for risk of diabetes incidence (22). Moreover, this ratio decreased in individuals following weight loss of 5% or more (22). A prospective study has revealed significant reductions in circulating very long chain ceramides (C20, C20:1, C22.1, C24, C26, C26:1) in type 1 diabetic patients that also associated with the development of nephropathy (23).

### Liver Disease

A clinical study consisting of 406 patients with chronic viral hepatitis revealed that levels of sphingosine, sphinganine, and certain ceramides significantly associated with the severity of liver fibrosis in HCV-infected patients (compared to HBV-infected patients) (24). Moreover, Apostolopoulou et al. demonstrated that elevation of various sphingolipids species in the liver and serum of NASH patients compared to non-alcoholic fatty liver and control subjects (25). Specifically, in NASH, hepatic dihydroceramides (16:0, 22:0, and 24:1) and lactosylceramides increased significantly. Total serum dihydroceramides and hepaic dihydroceramides (22:0 and 24:1) increased significantly in NASH and strongly associated with whole body insulin-resistance (25). Additional analysis shows that sphingolipid species correlated with hepatic oxidative stress and inflammation (25). In a prospective study consisting of 31 children diagnosed with non-alcoholic fatty liver disease (NAFLD), Wasilewska et al. demonstrated significant, positive correlation between total serum concentration of ceramides with insulin and also with HOMA-IR (26). Additionally, this study reveals that total ceramide concentration and specific (saturated fatty acyl) subspecies of ceramides such as C14, C16, C16:1, C18, and C18:1 were significantly higher in children with NAFLD compared to controls (26). Lastly, a randomized clinical trial conducted to determine the influence of dietary saturated and polyunsaturated fat on fatty liver development found that saturated fat markedly induces liver fat and serum ceramides (27). The effect was pronounced on C16 ceramides, whereas dietary polyunsaturated fat prevents liver fat accumulation and associated with reduced total ceramides (27).

### Bariatric Surgery

The benefits of bariatric surgery on obesity and metabolic disease are well-established, including an almost immediate remission of type 2 diabetes and hyperlipidemia. In this context, surgically induced weight loss, which was associated with the improvement in insulin sensitivity and a decrease in proinflammatory cytokines, was shown to decrease plasma ceramide levels. Numerous serum ceramides, including the C16 subspecies, decreased in a time-dependent manner post-surgery (28). Subsequently, another study reveals a significant, time-dependent reduction of serum C22 and C24 ceramides after laparoscopic sleeve gastrectomy (29).

Taken together, these clinical observations suggest that increases of saturated fatty acyl C16-18 ceramides, as well as several other sphingolipid species, are apparent in obese, fatty liver and insulin resistant individuals. The heterogeneity in species is a result of the dysregulated changes in ceramide biosynthesis in different organs. In this context, several mechanistic studies have found that the supply of saturated fat is sufficient to induce ceramides and cause metabolic dysfunction. For example, oversupply of saturated fatty acids by lipid infusion or diet promotes ceramide accumulation and activates inflammatory pathways, induces insulin resistance, impairs mitochondrial function, and stimulates endoplasmic stress and lipotoxicity. Blocking ceramide synthesis negates these saturated fatty acid actions (6, 8). Beyond saturated fatty acids, other factors, such as inflammatory signaling pathways, increase the rate of ceramide synthesis.

The above human lipodomic data (Table 1) also suggest that altered ratios of ceramide subspecies are associated with the comorbidities of diabetes and obesity. In this context, recent comprehensive sphingolipid analysis in rodent metabolic disease mouse models suggests that the ratio of long-chain ceramide species to very long-chain ceramide species in liver is a key marker of metabolic disease (32). Below I will discuss interventional studies in rodents suggest that accumulation of toxic species of ceramides such as C16 may play a role during progression of simple steatosis to NASH in humans.

**Table 1:** Altered ceramide ratios in insulin resistance, diabetes, and fatty liver disease.

**Altered circulating/tissue long and very long chain ceramide ratios associated with insulin resistance and diabetes**	
Neeland et al. (20)	Increased C16 and C18 ceramides in serum correlated with insulin resistance, total body fat, and visceral fat tissue
	Elevated levels of C24:2, C30:10, and C32:11 ceramides in serum associated with healthier metabolic profiles
Chew et al. (21)	Increased C16, C18, and C20 ceramides in serum correlated positively with body mass index (BMI) and HOMA-IR
Hilvo et al. (22)	Increased C18 ceramide in serum shows the strongest association with incident diabetes. Study identifies C18/16 ratios as an independent marker for risk of incidence of diabetes
Klein et al. (23)	Very long chain ceramides (C20, C20:1, C22.1, C24, C26, and C26:1) are significantly reduced in serum of type 1 diabetic
Bergman et al. (30)	Higher levels of C18 ceramide in skeletal muscle association with insulin resistance and inflammation
Perreault et al. (31)	C18-ceramide levels increased in the skeletal muscle cells isolated from individuals with type 2 diabetes
**Increased dihydroceramides/long chain ceramide with fatty liver diseases**	
Apostolopoulou et al. (25)	Total serum dihydroceramides and hepatic dihydroceramides (16:0, 22:0, and 24:1) increased in NASH Hepatic dihydroceramides (22:0 and 24:1) increased significantly in NASH and strongly associated with whole body insulin-resistance
Wasilewska et al. (26)	Serum saturated ceramides species such as C14, C16, C16:1, C18, and C18:1 significantly higher in children with NAFLD
Rosqvist et al. (27)	Dietary saturated fat markedly induces the fatty liver development, associated with increase serum total ceramides specifically pronounced effect observed in C16 ceramides Dietary polyunsaturated fat prevents fatty liver development associated with reduced serum total ceramides

## Cers Enzymes as Therapeutic Targets

The studies described above suggest that therapeutic interventions that reduce ceramides could have utility for treating insulin resistance and fatty liver disease. More recent efforts have interrogated the value of targeting the CerS enzymes to alter the acyl-composition of ceramides to treat the comorbidities of obesity. These interventions are producing promising results.

### CerS1 and Muscle Insulin Resistance

Skeletal muscle is the primary organ responsible for insulin-mediated glucose uptake and utilization. During obesity, excess energy is delivered to muscle in the form of non-esterified fatty acids (NEFA) or lipoprotein-bound triglycerides. Excess delivery of these fatty acids to skeletal muscle causes insulin resistance, as the muscles adapt to the use of more plentiful energy source. This condition increases one's risk for type 2 diabetes and cardiovascular disease (33). Mechanistic studies have suggested that diacylglycerols (34) and ceramides (6) may contribute to skeletal muscle insulin resistance development.

**Figure 2:**
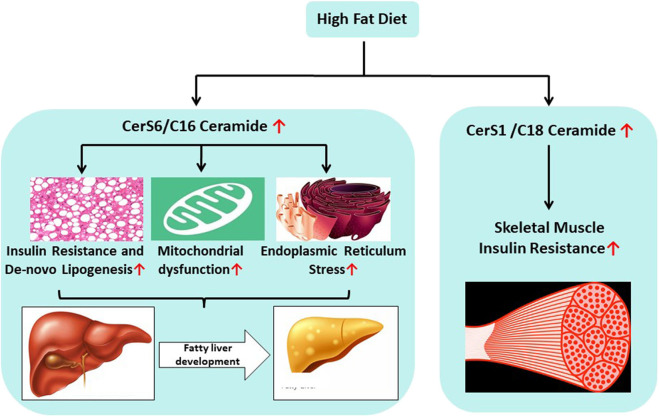
Unhealthy food habits promote the delivery of excess saturated fatty acids and carbohydrates that lead to increased biosynthesis of C16 and C18 ceramides in the liver and skeletal muscle, respectively. CerS6 mediated C16 ceramide plays critical role in the fatty liver development via (a) increased insulin resistance (b) increased *de-novo* lipogenesis, (c) mitochondrial dysfunction, and (d) increased endoplasmic reticulum stress. CerS1 mediated C18 ceramide is involved in the development of skeletal muscle insulin resistance.

CerS1 is the predominant isoform in muscle and the C18 ceramides that it produces are the major ceramide subspecies found in the tissue (9). Recent clinical observations identified higher levels of C18 ceramide in association with insulin resistance and inflammation in skeletal muscle (30). Another study found that C18-ceramide levels increased in skeletal muscle cells isolated from individuals with type 2 diabetes (31). To study the role of CerS1 in metabolic pathologies, researchers turned to rodent loss-of-function models (35). In these studies, high fat induced obesity led to an increase in C18 ceramides in muscle (35). Global ablation of CerS1 protected mice from high fat diet-induced weight gain. Moreover, the intervention increased energy expenditure, reduced adiposity, and improved insulin and glucose tolerance (35). Subsequently, the authors developed skeletal muscle-specific CerS1 knockout mice, demonstrating that tissue-specific ablation of the gene also improved glucose tolerance and insulin sensitivity (35). These observations suggest key roles for skeletal muscle C18 ceramides in the pathophysiology of obesity associated insulin resistance [Figure 2; (35)].

Turner et al. evaluated CerS1 as a potential drug target using novel small molecule inhibitors of the enzyme. The researchers developed P053, a specific inhibitor of CerS1 that displays nanomolar potency (36). PO53 displayed selectivity for CerS1 in mice by reducing C18 ceramide concentrations in skeletal muscle without changing other subspecies of ceramides (36). PO53-treated animals showed improved skeletal muscle mitochondrial beta-oxidation relative to vehicle treated group (36). However, PO53 treated animals did not show any improvement in glucose tolerance and insulin sensitivity, despite having decreased intramuscular C18 ceramides (by ~50%) (36). This is in apparent contrast to the studies using genetic knockouts, which markedly altered glucose disposal. The contradictory observations could relate to differences in the diet induced obese mouse models used, the duration of the study or the degree of CerS1 inhibition that was achieved: (a) C57BL/6N mice were used in the knockout study, while the PO53 study used smaller C57BL/6J mice; (b) the knockout animals were fed for 12 weeks while the PO53 study were treated for only 4–6 weeks; and (c) the PO53 pharmacological treatment had a more modest effect on ceramides, reducing them by ~50%, compared to >90% reduction in skeletal muscle of the knockout animals (35, 36). Therefore, further investigation of CerS1 inhibitors is warranted.

### CerS6 and NASH

NASH, characterized by hepatocellular lipid accumulation (steatosis) along with inflammation and varying degrees of fibrosis, is a serious condition affecting 1.5–6.45% of the population (1). Once afflicted, ~10–15% of NASH patients progress to cirrhosis (37). The estimated annual economic burden of NASH in the USA is $103 billion (38). NASH has no FDA-approved therapy, prompting many companies to race for approval of the first NASH-targeted drugs (38). Current disease management is primarily focused on promoting weight loss through lifestyle interventions, weight loss medication, and/or bariatric surgery (39). Limited prospective data are available on these options (39).

The pathology was originally interpreted to result from “dual-hits.” The first hit, the steatosis that characterizes NAFLD, predisposes individuals to the second hits that include inflammation (40). More recently, complex “multiple-hit” hypotheses have been proposed, as numerous other factors including oxidative stress, mitochondrial dysfunction, and other insults are implicated in the pathology (41). Each stage is defined by specific risk factors and pathological mechanisms (42). As noted above, a number of studies indicate that sphingolipids such as ceramides, which can induce each of these elements, including inflammation, oxidative stress, and mitochondrial dysfunction, to drive NASH (13). Myriocin, a potent inhibitor of SPT, inhibits NASH progression in rodents (43, 44).

Several studies suggest that altering ceramide acylation patterns through CerS inhibition could also alter disease progression. Mice lacking both copies of the CerS2 gene display numerous liver abnormalities such as hepatocyte death and chronic apoptosis and regeneration (45, 46). The animals develop hepatoadenoma at 3–4 months of age, markedly reduced body weight, and hepatocarcinoma (around age 1 year) (45, 46). Furthermore, homozygous CerS2 knockout mice display decreased lipid accumulation and uptake in the liver (47). These animals displayed the expected reduction in very long chain acyl ceramides; however, they had a compensatory increases in C16-ceramides (45, 46). This was interesting, as the C16-ceramides had been associated with hepatic insulin resistance (48) and disruption of the mitochondrial respiratory chain (49).

We subsequently studied heterozygous CerS2 knockout mice, which show a more modest pathology than their homozygous counterparts. The heterozygous CerS2 knockout mice show normal lifespan and no liver phenotype on normal chow diet (17) but phenotypes emerged when they were challenged with an obesogenic high fat diet (HFD). On the HFD, they displayed increased liver weight, triglycerides, macrophage infiltration, circulating liver enzymes, and plasma cholesterol, all of which are indicative of liver damage (17). These mice also displayed slightly impaired glucose tolerance, high fasting, and fed insulin levels, increased glucose mediated insulin secretion, reduced insulin sensitivity, decreased ambulatory activity, and increased fat to lean mass (17). As in the aforementioned study of the homozygous animals, the liver abnormalities were associated with reduced levels of very long chain ceramides coupled with the compensatory increase in C16-ceramides (17). In these studies, the increase in C16-ceramides could be explained by increased expression of CerS6. No changes were apparent on the normal chow diet. Mechanistic studies in primary hepatocytes obtained from the heterozygous CerS2 knockout mice revealed that the alteration in ceramide acylation attenuated insulin signaling and induced mitochondrial dysfunction (17). To recapitulate the compensatory increase of CerS6 expression, we overexpressed CerS6 in wild-type primary mouse hepatocytes. This intervention increased accumulation of C16 ceramides and compromised mitochondrial function, increased triglyceride accumulation and attenuated insulin signaling (17).

Additional studies in mice have shown that diet induced NAFLD is associated with liver ceramide acylation patterns that paralleled the profile of the CerS2 knockouts. In general, high fat diets lead to increases in CerS6 expression and a concomitant elevation of C16-ceramides (50). Of note, in one study, overexpression of CerS2 was protective, presumably because it prevented the induction of CerS6 (50). In that study, CerS6 was shown to induce sterol regulatory element binding protein-1 (SREBP-1) cleavage and decrease levels of INSIG-1, leading to increased *de-novo* lipogenesis (DNL) (50). SREBP-1 is a master regulator of DNL in the liver and one of the primary insults that is dysregulated in the NASH pathophysiology (42). These authors reproduced our work (17), showing that CerS2 heterozygotes were susceptible to diet-induced steatohepatitis, exhibiting a pronounced endoplasmic reticulum (ER) stress response (50). The involvement of ER-stress was novel, but not surprising; Cinar et al. previously demonstrated that HFD-induced hepatic insulin resistance was associated with increased ER stress that was associated with elevated hepatic C16 and C18 ceramides (51).

Bruning et al. obtained the strongest evidence to date that CerS6 was pathogenic. They demonstrated that levels of CerS6 were increased in visceral and subcutaneous adipose tissue of obese humans, correlating positively with BMI and insulin resistance (18). These authors also found that the CerS6 product C16 ceramides were elevated in the visceral adipose of obese humans (18). They subsequently created CerS6 null mice, which allowed for a precise determination of the role of the enzyme in the development of obesity and fatty liver disease. The CerS6 null mice had the expected reduction in C16 ceramides. On a non-obesogenic chow diet, they had no obvious phenotype. However, the CerS6 null mice were protected from HFD-induced obesity (18). The change in body mass was completely explained by a reduction in adiposity, including a decrease in adipocyte size, serum leptin, adipose macrophage infiltration, and adipose pro-inflammatory gene expression (18). Additionally, the CerS6 null mice displayed improved insulin sensitivity and glucose tolerance (18). Turpin et al. also produced tissue-specific knockout mice, excising the gene from macrophages, brown adipocytes, and liver (18). Deletion of CerS6 from the brown adipose depots reduced diet induced obesity and improved mitochondrial beta oxidation, leading to elevations in energy expenditure (18). Liver-specific CerS6 null mice displayed a partial protection from diet induced obesity but a robust protection from glucose intolerance and insulin resistance explained by enhanced insulin signaling relative to wild type animals (18). Deletion of CerS6 from macrophages had no effect (18).

Brüning's group later identified a novel molecular mechanism through which CerS6 derived C16:0 ceramides alter mitochondrial dynamics, determining that the lipids interacted with mitochondrial fission factor (52). Deficiency of either CerS6 or mitochondrial fission factor encoded by the Mff gene protected mice from fatty acid-induced mitochondrial fragmentation *in vitro*. Moreover, the two proteins genetically interacted *in vivo*, participating in a linear pathway that accounted for obesity-induced mitochondrial fragmentation (52). Of note, an independent study reported that germline CerS6 knockout mice suffer neurobehavioral defects (53). This developmental defect was not observed by Brüning's group, which did not report any abnormal behavioral side effects of CerS6 deletion (18). These differences could be possibly explained by different knockout mouse generation strategies that were used (18, 53). However, from the safety pharmacology prospective it may be advisable to restrict compound use during pregnancy and/or to develop compounds that spares central nerves systems (CNS).

To develop therapeutic approaches to inhibit CerS6, we tested whether selective ablation of CerS6 using antisense oligonucleotides (ASO) was sufficient to reverse metabolic abnormalities in mice that were made obese by high fat diet (DIO mice) or leptin deficiency (ob/ob mice) (54). Delivery of the CerS6 ASO selectively reduced CerS6 expression by 90%, predominantly in the liver. CerS6 knockdown reduced C16:0 ceramides by about 50% in both liver and plasma (54). CerS6 ASO treatment efficiently lowered body weight gain and reduced body fat and fed (and fasted) blood glucose levels (1% reduction in HbA1c). Moreover, CerS6 inhibition improved oral glucose tolerance and insulin sensitivity (54). Both genetic knockout and ASO mediated genetic knock-down studies clearly demonstrated that inhibition of CerS6 activity ameliorated metabolic diseases including insulin resistance, type 2 diabetes and obesity [Figure 2; (17, 18, 54)]. Therefore, further discovery of selective small molecule therapeutics targeting CerS6 inhibition is warranted.

### CerS5 and Obesity

Gosejacob et al. developed CerS5 knockout mice and demonstrated that CerS5 null mice are viable and fertile and do not show any obvious morphological and phenotypic alterations on normal chow diet (55). However, when challenged with high fat diet CerS5 knockout animals protected against diet induced obesity and associated with reduced levels of leptin relative to wild type animals (55). Additionally, on diet induced obesity CerS5 knockout animals displayed improved glucose tolerance, insulin sensitivity, and reduced white adipose inflammation compared to wild type animals (55). This was also in contrast to the Brüning study, which showed no effect of CerS5 depletion (52).

## Conclusions

Clinical observations and experimental studies in rodents suggest that specific ceramides have distinct roles in the pathophysiology of various human diseases. Studies reveal that altering sphingolipid acylation patterns impacts hepatic steatosis, adiposity, adipocyte size, adipokine secretion, macrophage infiltration, inflammation, insulin sensitivity, mitochondrial dysfunction, and ER stress. In particular, the ratio of long-chain ceramides (e.g., C16 and C18) to very long-chain ceramides (e.g., C24:0 or C24:1) appears to be a key factor in the development of metabolic disease. Therefore, inhibition of CerS6, and perhaps CerS1 and CerS5, may serve as an attractive therapeutic approach for treating insulin resistance, obesity, fatty liver, and NASH.

## Author Contributions

SR researched the literature and wrote the manuscript.

## Conflict of Interest

SR is an employee of Evotec International GmbH, Germany.
